# Senescence and Inflammatory Markers for Predicting Clinical Progression in Parkinson’s Disease: The ICICLE-PD Study

**DOI:** 10.3233/JPD-191724

**Published:** 2020-01-13

**Authors:** Carmen Martin-Ruiz, Caroline H. Williams-Gray, Alison J. Yarnall, John J. Boucher, Rachael A. Lawson, Ruwani S. Wijeyekoon, Roger A. Barker, Claire Kolenda, Craig Parker, David J. Burn, Thomas Von Zglinicki, Gabriele Saretzki

**Affiliations:** a The NIHR Newcastle Biomedical Research Centre, Newcastle University, Campus for Ageing and Vitality, Newcastle Upon Tyne, UK; b Translational and Clinical Research Institute, Newcastle University, UK; c Department of Clinical Neurosciences, John Van Geest Centre for Brain Repair, University of Cambridge, Cambridge, UK; d The Newcastle upon Tyne Hospitals NHS Foundation Trust (NUTH), Newcastle Upon Tyne, UK; e Biosciences Institute, Newcastle University, Campus for Ageing and Vitality, Newcastle Upon Tyne, UK; f Current address: Department of Neurology, University College Hospital, Galway, Ireland; g WT-MRC Cambridge Stem Cell Institute, University of Cambridge, Cambridge, UK; h Population Health Sciences Institute, Faculty of Medical Sciences, Newcastle University, The Newcastle upon Tyne Hospitals NHS Foundation Trust (NUTH), Newcastle Upon Tyne, UK

**Keywords:** Parkinson’s disease, telomere length, senescence, p16, p21, inflammation, biomarker, cognitive impairment, dementia

## Abstract

**Background:**

Cognitive decline is a frequent complication of Parkinson’s disease (PD) and the identification of predictive biomarkers for it would help in its management.

**Objective:**

Our aim was to analyse whether senescence markers (telomere length, p16 and p21) or their change over time could help to better predict cognitive and motor progression of newly diagnosed PD patients. We also compared these senescence markers to previously analysed markers of inflammation for the same purpose.

**Methods:**

This study examined the association of blood-derived markers of cell senescence and inflammation with motor and cognitive function over time in an incident PD cohort (the ICICLE-PD study). Participants (154 newly diagnosed PD patients and 99 controls) underwent physical and cognitive assessments over 36 months of follow up. Mean leukocyte telomere length and the expression of senescence markers p21 and p16 were measured at two time points (baseline and 18 months). Additionally, we selected five inflammatory markers from existing baseline data.

**Results:**

We found that PD patients had shorter telomeres at baseline and 18 months compared to age-matched healthy controls which also correlated to dementia at 36 months. Baseline p16 levels were associated with faster rates of motor and cognitive decline over 36 months in PD cases, while a simple inflammatory summary score at baseline best predicted cognitive score over this same time period in PD patients.

**Conclusion:**

Our study suggests that both inflammatory and senescence markers (p16) are valuable predictors of clinical progression in PD patients.

## INTRODUCTION

It is well established that the cumulative incidence of dementia associated with Parkinson’s disease (PD) is approaching 80% [1–3] and individuals with PD are five to six times more likely to develop cognitive impairment than age-matched controls [4]. Dementia limits the effective medical and surgical management of motor features of PD and is associated with increased mortality [5], reduced quality of life [6] and greater caregiver distress [7].

The causes for dementia in PD (PDD) are heterogeneous, complex and not fully understood. The development of biological markers that could identify those at highest risk of early cognitive decline would enhance our understanding of disease progression and give valuable insights into the underlying pathophysiological mechanisms.

Telomeres are protective structures at chromosomal ends. Telomere shortening and dysfunction cause cellular senescence [8]. Importantly, telomeres are susceptible to oxidative stress [9] and can be indicators of ongoing disease processes in the brain. We have previously shown that short leukocyte telomeres predict the development of cognitive decline and vascular dementia in stroke patients [10]. However, contradicting results exist regarding a possible correlation between telomere length in leukocytes and PD [11–13].

We hypothesised that accelerated telomere shortening in the early stages of PD may predict cognitive decline. This is based on the findings that in individuals without dementia, performance on specific subsets of cognitive tests correlates positively with leukocyte telomere length [14, 15], and cognitive decline over time is slower in subjects with longer telomeres [16, 17]. Studies assessing the association between blood telomere length and incident dementia yielded variable and contradicting results [18–20]. Similarly, for PD there are conflicting results on the association between telomere length and PD incidence [12, 13, 21, 22] or progression towards cognitive decline and dementia [23–26]. A recent meta-analysis concluded that there was no association between leukocyte telomere length and incidence of PD [11]. However, the relationship between telomere length or telomere attrition rates and the progression of motor and cognitive aspects of PD has not been extensively investigated.

Cellular senescence is a response to stress (including shortened or damaged telomeres) characterized by an irreversible cell cycle arrest, a pro-inflammatory secretory phenotype and a number of not entirely specific tumour-suppressor proteins, including the cyclin-dependent kinase inhibitors p21^CIP1Waf1^ and p16^INK4A^ [27]. A meta-analysis of more than 300 genome wide association studies (GWAS) identified the p16^INK4a^ gene to be linked to the highest number of age-associated pathologies, including several types of cardiovascular diseases, diabetes, glaucoma and AD [28]. Consequently, p16 and p21 have been investigated as cell senescence-associated biomarkers of ageing [29, 30]. Brain ageing, neurodegeneration and neuroinflammation are associated with cellular senescence of astrocytes, oligodendrocytes, microglia and neurons [31–34]. In mice as well as in humans, the neuronal accumulation of neurofibrillary tangles associated with cognitive decline and neuronal loss in Alzheimer’s disease causes a cellular senescence phenotype in the affected neurons [35]. In a tau-related mouse model, specific eradication of senescent cells improved cognition and decreased neurodegeneration [36]. In humans, the potential of both p16 and p21 expression as biomarkers of ageing and age-related diseases has previously been explored [37–39] although not yet in the context of PD.

A previous study on the Incidence of Cognitive Impairments in Cohorts with Longitudinal Evaluation-Parkinson’s Disease (ICICLE-PD) concluded that the serum inflammatory marker profile measured at the time of diagnosis is associated with subsequent rate of disease progression [40]. A pro-inflammatory cytokine profile was associated with more rapid motor decline as defined by changes in UPDRS-III score over 36 months of follow-up, as well as lower cognitive scores, whereas an anti-inflammatory profile was associated with stable motor function over time and better cognition [40]. Given the close inter-connection between oxidative stress, cell senescence and inflammation as the main drivers of the ageing process and contributors to age-related diseases [41, 42], our aim was to determine whether there is an association between markers of cell senescence and cognitive and motor function in PD patients over the first three years of their disease. For this purpose, we analysed telomere dynamics and senescence modulators in newly diagnosed patients and controls from the ICICLE-PD cohort and combined this with existing data on baseline inflammatory markers to explore the relevance of these factors as predictors of cognitive and motor impairment over a period of 36 months from baseline. We hypothesised that increased senescence and inflammation at baseline would predict faster cognitive decline during disease progression in the early stages of disease.

## MATERIALS AND METHODS

### Participants and clinical evaluation

Details of the recruitment protocol for the study have been published [6, 43, 44]. Participants were recruited as part of the ICICLE-PD study between June 2009 and December 2011. They included newly diagnosed PD patients (N = 154) from the community and outpatient clinics, as well as unrelated age and gender—matched control subjects (N = 99) from Newcastle upon Tyne and Gateshead, United Kingdom. All patients fulfilled Queen Square Brain Bank Criteria for idiopathic PD [45] and were diagnosed by a movement disorder specialist. Global assessment of cognitive function included the Mini-Mental State Examination (MMSE) [46] and Montreal Cognitive Assessment (MoCA) [47]. Exclusion criteria have been described previously [43]. The study received ethical approval from The Newcastle and North Tyneside Research Ethics Committee and written informed consent was received from all subjects.

Participants were evaluated at 18-month intervals. At each assessment, demographic, cognitive and clinical data were collected as well as blood samples. For the clinical assessment, a semi-structured interview collected information on areas such as symptom history, level of education and comorbid disease. Motor severity was assessed using the Movement Disorder Society Unified Parkinson’s Disease Rating Scale (MDS-UPDRS) Part III [48] and the Hoehn and Yahr (HY) scale [49]. Data on medication usage included levodopa equivalent daily dose (LEDD) at the time of recruitment [50]. Mild Cognitive Impairment (PD-MCI) was assessed using modified MDS Level 2 criteria [51], whereby PD-MCI was classified using 1.5 SDs below normative values [44].

### Blood sample collection

For inflammatory markers, venous blood samples were collected in plain tubes, allowed to clot for 15 min at room temperature, and then centrifuged at 2000 rpm for 15 min. Serum was removed and stored in 200*μ*L aliquots at –80°C until assays were performed. For the isolation of DNA, blood samples were collected using EDTA vacutainers (Becton Dickinson, BD Diagnostics, Oxford, UK). For the isolation of RNA, blood samples were collected in PAXgene Blood RNA Tubes (PreAnalytiX, Qiagen, UK). The collection tubes were stored at –80°C.

### Nucleic acid isolation

DNA was extracted from peripheral blood using standard phenol/chloroform techniques. DNA sample concentration and quality was assessed by Quant-It™Picogreen^®^ (ThermoFisher Scientific, USA) and stored at –80°C.

RNA isolation was performed using the PAXgene Blood RNA Kit (PreAnalytiX, Qiagen UK) according to the manufacturer’s instructions. The final elution volume was 50*μ*l and the quantity and quality of the RNA was assessed by Nanodrop (ND-1000, Thermo Fisher Scientific, USA) followed by storage at –80°C.

### Telomere length measurement

Telomere length (TL) was measured using a previously published quantitative PCR (qPCR) method with modifications [52]. Briefly, the relative abundance of telomeric template (T) versus a single copy gene (36B4) (S) was estimated by PCR on 10 ng of DNA, with 5ul SYBR^®^Green JumpStart Taq Ready Mix and 0.25*μ*l of ROX reference dye (Sigma-Aldrich, UK) and the following primers: 300 nM TelA (5′-CGG TTT GTT TGG GTT TGG GTT TGG GTT TGG GTT TGG GTT-3′); 900 nM TelB (5′-GGC TTG CCT TAC CCT TAC CCT TAC CCT TAC CCT TAC CCT-3′) for the telomeric reaction and 200 nM 36B4F (5′-CAG CAA GTG GGA AGG TGT AAT CC 3′) and 400 nM 36B4R (5′-CCC ATT CTA TCA TCA ACG GGT ACA A-3′) for 36B4. All samples were assessed in triplicate and all PCRs were carried out on an Applied Biosystems 7900HT Fast Real Time PCR system with 384-well plate capacity. Telomere length measurements are expressed as T/S ratio. Three internal control DNA samples of known telomere length (10.4 kb, 3.9 kb, and 2 kb) were run within each plate to correct for plate-to-plate variation. As a further authentication of our telomere length measurements, we performed a revaluation of those samples that were in either the top or bottom 5% of the telomere length distribution as well as those samples that gave no valid data on the first run. The intra-assay coefficient of variation was 2.7% while the inter-assay coefficient of variation was 5.1%. DNA samples were available to perform telomere length measurements at baseline and at 18-month follow-up visit.

### Expression of p16 and p21

The quantification of expression levels of p21 and p16 was performed by RT-qPCR analysis on an Applied Biosystems 7900HT Fast Real Time PCR system with 384-well plate capacity, with 500 ng of RNA converted into cDNA by means of the qScript cDNA synthesis kit (Quanta Biosciences, Beverley, MA, USA) on a 20*μ*l reaction that included 1ul of qScript reverse transcriptase. The reaction comprised 5 min at 22°C, 30 min at 42°C and 5 min at 85°C. This was followed by qPCR on p16 and p21 with PGK1 as reference gene: the 15*μ*l reactions included 2*μ*l of cDNA, 7.5*μ*l of Applied Biosystems Power SYBR™ Green PCR Master Mix (ThermoFisher Scientific, Waltham, MA, USA) and 300 nM for both forward and reverse primers for the respective reactions: p16-1 5′-GCCCAACGCACCGAATAGTT -3′ and p16-2 5′-CACGGGTCGGGTGAGAGT -3′; p21-F 5′-GCAGACCAGCATGACAGATTT -3′ and p21-R 5′-GGATTAGGG CTTCCTCTTGGA -3′; and PGK1-1 5′-TTAAAGGGAAGCGGGTCGTT -3′ and PGK1-2 5′-GCAGCCTTAATCCT CTGGTTGT -3′. For each sample, all three qPCR reactions (p21, p16 and PGK1) were performed in triplicate and on the same plate. The thermo-cycling programme included 10 min at 95°C and 45 cycles on 15s at 95°C and 30s at 60°C as well as a melting curve analysis. Each plate included an Internal Control cDNA sample to correct for plate-to-plate variations. The intra-assay % CV for the p16, p21 and PGK1 qPCR reactions were 0.60%, 0.65%, and 0.91%, respectively, while the inter-assay % CV for the p16, p21 and PGK1 qPCR reactions were 3.80%, 2.55%, and 3.82%, respectively. Values of expression levels for p16 and p21 were calculated by the formula 2^–*ΔΔ*Ct^ on the differences on threshold cycle for p16 or p21 against PGK1 and for the samples against those obtained for the internal control. RNA samples were available to perform p21 and p16 gene expression measurements at baseline and at 18-month follow-up visit.

### Inflammatory markers

Measurement of inflammation-related markers on serum samples from this cohort has been previously performed and reported [40]. Briefly, markers were measured on serum samples collected at baseline, using MesoScale Discovery (Rockville) electrochemiluminescent immunoassays, including the V-PLEX human pro-inflammatory panel 1 (IFN-*γ*, IL-1*β*, IL-2, IL-4, IL-6, IL-8, IL-10, IL-12p70, IL-13, and TNF-*α*), and V-PLEX human CRP. All samples were run in duplicate and as per the manufacturer’s protocol [40] which yields the following values for the inflammatory markers considered in our analysis: Inter- assay % CVs for CRP: 7.2%; TNF-alpha: 7.4%; IL–6:6.8%; IL–10:7.9% and IFN-gamma: 8.2%. Intra-assay % CVs for CRP: 2.2%; TNF-alpha: 2.4%; IL–6:3.9%; IL–10:3.1% and IFN-gamma: 3.8%. Lower Limit of Detection for CRP: 1.33 pg/ml; TNF-alpha: 0.04 pg/ml; IL–6:0.06 pg/ml; IL–10:0.04 pg/ml and IFN-gamma: 0.37 pg/ml. From this existing data set, five pro-inflammatory markers (TNF-alpha, IFN-gamma, CRP, IL-6 and IL-10) were selected because they had the strongest loadings on the pro-inflammatory component scores in our previous principal component analysis (PCA) in this ICICLE-PD dataset [40]. In this study we sought to validate the findings of Williams-Gray et al. [40] but through the use of a smaller number of parameters combined into a simple composite score, which does not require a PCA-based approach.

### Statistical analyses

Statistical analyses were performed with SPSS 19.0 (SPSS, Chicago, IL). Data were examined for normality of distribution (Shapiro-Wilk test and Q-Q plots). Baseline characteristics are expressed as the median [Interquartile Range] or as percentages. The demographic characteristics of our participants were analysed by either an Independent samples Mann-Whitney U test (for continuous variables) or Pearson Chi-square test (for categorical variables). The percentage of participants for which data were unavailable (either because the clinical information was not acquired or because the suitable blood samples were not collected) appear in the column “Missing”. Because of their lack of normal distribution, data were analysed by Spearman’s rank correlation coefficient (for correlations between blood-related biomarkers of senescence and outcome measures, correlations were adjusted for age, gender and baseline LEDD) and Mann-Whitney U tests (for comparisons between Parkinson disease and control participants). Pearson-Chi squared tests were used to compare categorical data. Results were considered statistically significant at a *p* value of <0.05 and two-sided tests were applied.

Composite variables were calculated as detailed below:

- Telomere length change per month:

$$\frac{\left(\mathrm{TL}\mathrm{at}18\mathrm{month}\mathrm{Follow}-\mathrm{up}\right)-\left(\mathrm{TL}\mathrm{at}\mathrm{Baseline}\right)}{\mathrm{Interval}\mathrm{between}18\mathrm{month}\mathrm{Follow}-\mathrm{up}\mathrm{and}\mathrm{Baseline}\;\left(\mathrm{in}\;\mathrm{months}\right)}$$

A more negative value of this variable indicates faster telomere shortening.

- p21 expression change per month:

$$\frac{\left(p21\;\mathrm{Expression}\mathrm{at}18\mathrm{month}\mathrm{Follow}-\mathrm{up}\right)-\left(p21\mathrm{Expression}\mathrm{at}\mathrm{Baseline}\right)}{\mathrm{Interval}\mathrm{between}18\mathrm{month}\mathrm{Follow}-\mathrm{up}\mathrm{and}\mathrm{Baseline}\;\left(\mathrm{in}\;\mathrm{months}\right)}$$

A more positive value for this variable reflects a transition to a more senescent phenotype.

- p16 expression change per month:

$$\frac{\left(p16\mathrm{Expression}\mathrm{at}18\mathrm{month}\mathrm{Follow}-\mathrm{up}\right)-\left(p16\;\mathrm{Expression}\mathrm{at}\mathrm{Baseline}\right)}{\mathrm{Interval}\mathrm{between}18\mathrm{month}\mathrm{Follow}-\mathrm{up}\mathrm{and}\mathrm{Baseline}(\mathrm{in}\mathrm{months})}$$

A more positive value for this variable reflects a transition to a more senescent phenotype.

- Inflammatory score: it was generated from the sum of the Z-scores for the inflammatory parameters offering the most comprehensive dataset: CRP, TNF-alpha, IL-6, IL-10 and Interferon-gamma (IFN-gamma).

- Monthly change rate of cognitive and clinical PD scores:

$$\frac{\left(\mathrm{Score}\;\mathrm{at}36\mathrm{months}\mathrm{follow}-\mathrm{up}\right)-\left(\mathrm{Score}\mathrm{at}\mathrm{Baseline}\right)}{\mathrm{Interval}\mathrm{between}36\mathrm{months}\mathrm{Follow}-\mathrm{up}\mathrm{and}\mathrm{Baseline}\;\left(\mathrm{in}\mathrm{months}\right)}$$

For MMSE and MoCA, a more negative value represents a greater decline in cognition. Conversely, scores for clinical features (MDS-UPDRS-III and H&Y) increase with severity and therefore a larger positive value of the rate of change represents a more rapid worsening of their clinical condition. In order to be able to identify as many significant comparisons as possible while still maintaining a low false positive rate we have applied the Benjamini-Hochberg multiple comparisons correction with a 5% false discovery rate.

We used Linear Regression models to assess the relative value of our biomarker variables to predict the changes with time (36 months) in cognitive and motor parameters in our PD participants only. These analyses were adjusted for age, gender, BMI and baseline LEDD. We used baseline predictors of cognitive change and therefore did not account for increase in LEDD between baseline and 36 months in this analysis. A previous publication from our group had demonstrated that LEDD did significantly increase over time, however, it was not associated with change in cognitive scores [53].

## RESULTS

### Study demographics

The demographic characteristics of our study group at baseline (99 controls and 154 PD patients) are described in Table 1. The table shows the study groups were well matched for most of the demographic and clinical characteristics. The group of PD participants presented a significantly lower alcohol consumption, higher proportion of cases with previous history of stroke or ischaemic attack and, as anticipated, higher presence of MCI, lower MoCA scores and higher Geriatric Depression Scale (GDS) scores.

**Table 1:** Baseline clinical characteristics of the study cohort

	Parkinson’s Disease		Controls	
	N = 154		N = 99	
		Missing		Missing	*p*
Age (Years, Median [IQR])	67.02 [60.28,81.50]	–	67.98 [63.34,82.15]	–
Gender (% Women)	35.06%	–	45.45%	–
Body Mass Index (Median [IQR])	26.50 [23.80,35.60]	1.30%	27.70 [24.70,34.50]	2.02%
Smoking (% Never smoked)	55.19%	–	50.51%	–
Age when leaving education (Years, Median [IQR])	16.00 [15.00,25.00]	–	17.00 [15.00,23.00]	–
Number of units of alcohol consumed per week (Median [IQR])	3.50 [0.00,24.00]	–	6.00 [1.00,60.00]	–	*p* = 0.019^a^
Ischemic heart disease (%)	11.04%	–	9.09%	–
Type II diabetes (%)	7.79%	–	5.05%	–
Hypertension (%)	31.17%	–	35.35%	–
Hypercholesterolaemia (%)	11.69%	–	20.20%	–
Stroke/Transient Ischaemic Attack (%)	7.14%	–	0.00%	–	*p* = 0.007^b^
Anti-inflammatory medication (%)	33.33%	12.34%	33.33%	6.06%
Comorbidity (CIRS System Score, Median [IQR])	2.00 [1.00,5.00]	12.34%	3.00 [2.00,6.00]	6.06%
Mild Cognitive Impairment (%)	43.26%	8.44%	22.22%	–	*p* < 0.001^b^
Mini-Mental State Examination Score (Median [IQR])	29.00 [28.00,30.00]	–	29.00 [28.00,30.00]	–	*p* = 0.006^a^
Montreal Cognitive Assessment (Median [IQR])	26.00 [23.00,30.00]	9.74%	27.00 [26.00,30.00]	2.02%	*p* < 0.001^a^
Geriatric Depression Scale (Median [IQR])	2.00 [1.00,8.00]	–	1.00 [0.00,4.00]	–	*p* < 0.001^a^
National Adult Reading Test (Median [IQR])	117.00 [108.50,127.00]	1.30%	118.00 [111.00,126.00]	–
Survival at 36months Follow-up (%)	94.81%	–	97.98%	–
Disease Duration at Enrolment (Years, Median [IQR])	0.39 [0.21,1.32]	–	–	–
MDS-UPDRS-III (Median [IQR])	26.00 [17.00,47.00]	–	–	–
Hoehn and Yahr Score (HY) (Median [IQR])	2.00 [2.00,3.00]	–	–	–
Parkinson’s disease medication (%)	87.66%	–	–	–
On Levodopa treatment (%)	29.22%	–	–	–
Levodopa equivalent daily dose (mg/l) (Median [IQR])	140.00 [100.00,450.00]	–	–	–
Monoamine oxidase B inhibitors medication (%)	47.40%	–	–	–
Dopamine agonist medication (%)	37.01%	–	–	–

^a^Independent-Samples Mann-Whitney U Test; ^b^Pearson Chi-square test.

### Biomarkers of senescence and PD

We measured three biomarkers of cell senescence: expression levels of p21 and p16 as well as telomere length, at baseline, and again at 18 months, which allowed for calculation of monthly rates of change (Table 2).

**Table 2:** Biomarkers: comparison of controls and PD patients

	Parkinson’s Disease		Controls	
	N = 154		N = 99	
	Median [IQR]	Missing	Median [IQR]	Missing	*p*
Telomere length (TL) Baseline (T/S ratio)	0.78 [0.63,0.91]	20.78%	1.03 [0.81,1.31]	22.22%	*p* < 0.001^a^
TL change/month^†^	–0.020 [–0.028,–0.013]	20.78%	–0.012 [–0.023,–0.006]	22.22%	*p* = 0.002^a^
p21 Baseline (A.U.)	1.16 [0.83,1.73]	6.49%	1.62 [1.01,2.43]	6.06%	*p* < 0.001^a^
p21 change/month	0.002 [–0.028,0.046]	20.78%	–0.006 [–0.044,0.036]	23.23%
p16 Baseline (A.U.)	1.24 [0.77,1.92]	6.49%	1.41 [0.94,1.97]	5.05%
p16 change/month	0.005 [–0.031,0.045]	20.78%	–0.001 [–0.032,0.043]	23.23%
CRP (ng/ml)	1.63 [0.66,3.48]	12.34%	1.83 [0.73,3.59]	6.06%
TNF alpha (pg/ml)	2.98 [2.45,3.62]	12.34%	1.67 [1.39,2.11]	6.06%	*p* < 0.001^a^
IL-6 (pg/ml)	0.64 [0.43,0.99]	13.64%	0.60 [0.42,0.81]	6.06%
IL-10 (pg/ml)	0.24 [0.17,0.35]	16.23%	0.19 [0.13,0.27]	12.12%	*p* = 0.002^a^
IFNg (pg/ml)	5.92 [4.11,8.80]	12.34%	5.80 [4.32,9.03]	6.06%
Inflammatory score	0.01 [–0.84,1.26]	17.53%	–1.26 [–1.85,–0.17]	12.12%	*p* < 0.001^a^

^a^Independent-Samples Mann-Whitney U test significant after Benjamini-Hochberg multiple test correction. ^†^For this composite variable, larger value = lower telomere shortening rate, see text.

**Fig.1:**
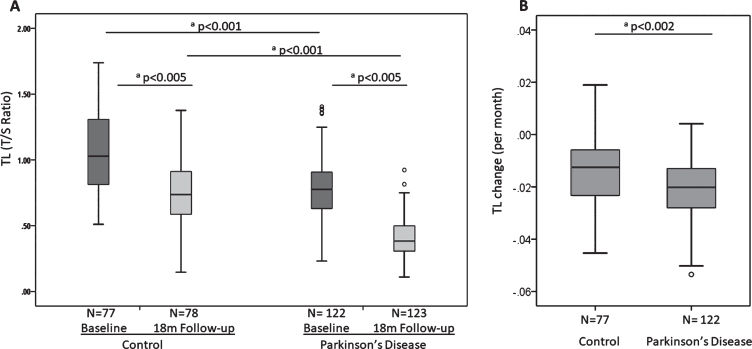
Telomere length at baseline and 18 months (A) and telomere shortening (given as change per months) over 18 months (B) in controls and Parkinson’s disease patients. Each category box plot includes the median (**—**), the range of data within the first and third quartiles (box), the range of data within the first and ninth deciles (whiskers) and the outliers falling outside of the latter (°). ^a^Statistically significant using the Mann-Whitney U Test.

After applying Benjamini-Hochberg correction for multiple comparisons, PD participants had significantly shorter telomere length at baseline compared to controls (*p* < 0.001) as well as significantly faster shortening of their telomeres over the first 18 month period (*p* = 0.002) (Table 2, Fig. 1). PD participants displayed significantly lower levels of p21 expression than controls at baseline (*p* < 0.001), but there was no difference in change with time (Table 2, Supplementary Figure 1a). For p16 expression, the differences between PD and controls at baseline or the rate of change per month of p16 expression levels did not reach statistical significance (Table 2, Supplementary Figure 1b).

### Inflammatory markers at baseline and PD

We considered five inflammatory markers, both individually and as a composite inflammatory score (Table 2). PD participants had significantly higher levels of TNF-alpha (*p* < 0.001) and IL-10 (*p* = 0.002) compared to controls. Overall, the baseline composite inflammatory score was markedly different between both groups, with a significant difference (*p* < 0.001) between PD and controls (Table 2).

### Biomarkers of senescence and inflammation: MCI at baseline

We examined whether our potential biomarkers were associated with MCI status. For the PD group (Table 3), of the five inflammatory markers considered, levels of CRP and IL-6 were significantly raised (*p*-values 0.015 and 0.003, respectively) in PD-MCI cases compared to those without cognitive impairment, and the composite inflammatory score of PD-MCI participants at baseline was significantly higher (*p* = 0.006) (Table 3). We found p21 expression levels also higher in cognitively normal participants than in those with PD-MCI at baseline (*p* = 0.012). No significant difference was found for telomere length or p16 expression in relation to PD-MCI (Table 3). In the case of participants from the control group, the presence of MCI did not result in significantly different levels of any of the senescence or inflammatory biomarkers (data not shown).

**Table 3:** Biomarkers and MCI at baseline in PD participants

	Cognitively Normal		MCI at Baseline	
	N = 80		N = 61		*p*
	Median [IQR]	Missing	Median [IQR]	Missing
TL Baseline (T/S ratio)	0.77 [0.58,0.90]	21.25%	0.79 [0.64,0.93]	22.95%
p21 Baseline (A.U.)	1.34 [0.93,1.98]	6.25%	1.03 [0.74,1.49]	4.92%	*p* = 0.012^a^
p16 Baseline (A.U.)	1.14 [0.74,1.74]	6.25%	1.29 [0.77,1.80]	4.92%
CRP (ng/ml)	1.10 [0.41,2.85]	12.50%	2.20 [0.82,5.26]	14.75%	*p* = 0.015^a^
TNF alpha (pg/ml)	2.74 [2.21,3.41]	12.50%	3.25 [2.51,3.78]	14.75%
IL-6 (pg/ml)	0.58 [0.36,0.87]	15.00%	0.78 [0.58,1.34]	14.75%	*p* = 0.003^a^
IL-10 (pg/ml)	0.23 [0.17,0.35]	17.50%	0.26 [0.17,0.39]	16.39%
IFNg (pg/ml)	5.78 [4.18,8.04]	12.50%	6.46 [4.88,11.23]	14.75%
Inflammatory score	–0.27 [–0.89,0.54]	20.00%	0.51 [–0.44,2.24]	16.39%	*p* = 0.006^a^

^a^Independent-Samples Mann-Whitney U test significant after Benjamini-Hochberg multiple test correction. ^†^For this composite variable, larger value = lower telomere shortening rate, see text.

### Biomarkers of senescence and inflammation: Dementia incidence at 36 months

**Fig.2:**
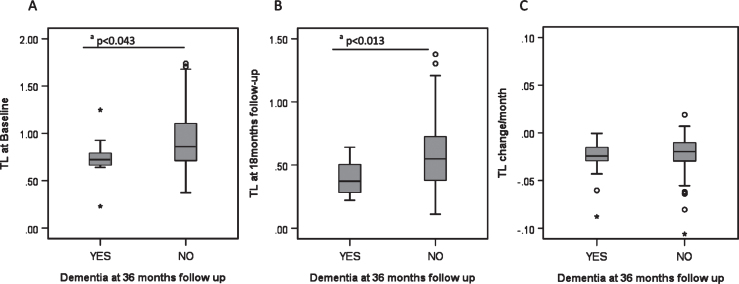
Biomarkers and development of dementia in PD. A) Telomere length at baseline and its relationship to PD dementia at 36 months. B) Telomere length at 18 month follow-up and its relationship to PD dementia at 36 months. C) Telomere length shortening per month and its relationship to PD dementia at 36 months. Each category box plot includes the median (**—)**, the range of data within the first and third quartiles (box), the range of data within the first and ninth deciles (whiskers) and the outliers falling outside the latter (° = Outlier, ^*^ = Extreme Outlier). ^a^Statistically significant using the Mann-Whitney U Test.

Over the 36 month follow-up period, 15.5% (N = 11) of PD patients developed PDD. These participants had significantly shorter telomeres at baseline and 18 months than those who did not develop PDD (Fig. 2) but there were no significant differences for p21, p16 or inflammatory score at baseline or 18 months follow-up (data not shown).

### Biomarkers of senescence and inflammation as predictors of cognitive and motor changes over time

We first examined the associations between senescence and inflammatory markers at baseline with cognition (MMSE and MoCA) and motor severity (MDS-UPDRS-III) at both baseline and 36 months by Spearmann’s Rank correlation analysis, and found correlations between senescence and inflammation markers and multiple clinical variables in PD patients (Supplementary Table 1). We analysed the predictive power of baseline biomarkers further by linear regression (Table 4). For the assessment of motor impairment, we regarded HY scores as a binary variable with a threshold of 3 (a score of 3 or above is indicative of postural instability). We considered both baseline, 36 months and the change between them and tested associations of baseline biomarkers of senescence and inflammation to these scores (data not shown). This was followed by a logistic regression model to determine whether our biomarkers at baseline would distinguish PD participants reaching HY stage 3 or above at the 36 month follow-up time and/or whether they discriminated between participants whose motor function improved or worsened over this period of time (Table 5). Neither TL nor p21 expression significantly predicted clinical scores or their change over 36 months (Tables 4 and 5). Paradoxically, higher p16 expression levels at baseline predicted slower motor progression (change of MDS-UPDRS-III score) over the next 36 months, independently of age and gender, baseline LEDD and BMI (beta = –0.218; *p* = 0.0149) (Table 4) and remained significant after adjusting for inflammatory score (beta = –0.226; *p* = 0.019, data not shown). In addition, higher p16 expression levels at baseline predicted slower cognitive decline (MoCA score change rate) over 36 months, independently of age, gender, baseline LEDD and BMI (beta = 0.311; *p* = 0.003) (Table 4). This association again remained significant after adjusting for inflammation (beta = 0.399; *p* < 0.001, data not shown).

**Table 4:** Linear regression modelling on biomarkers and disease progression in Parkinson’s disease participants^*^

Baseline Biomarkers		TL (T/S ratio)		p21 expression (A.U.)		p16 expression (A.U.)		Inflammatory Score	
		Beta	*p* value	Beta	*p* value	Beta	*p* value	Beta	*p* value
MDS-UPDRS-III	at 36 months	–0.068	0.477	0.021	0.811	–0.175	0.051	0.174	0.079
	change rate over 36 months	–0.020	0.832	0.110	0.216	–0.218	**0.014**	–0.004	0.970
MMSE	at 36 months	0.149	0.134	–0.009	0.922	0.080	0.409	–0.068	0.512
	change rate over 36 months	0.043	0.682	–0.031	0.751	0.120	0.231	0.116	0.287
MoCA	at 36 months	0.106	0.262	0.008	0.934	0.080	0.389	–0.205	**0.037**
	change rate over 36 months	0.066	0.560	–0.070	0.516	0.311	**0.003**	0.055	0.653

^*^All models adjusted for age, gender, baseline LEDD and BMI.

**Table 5:** Logistic Regression modelling on biomarkers and motor impairment (HY 3 or above) at 36 months follow-up

Baseline Biomarkers			TL (T/S ratio)		p21 expression (A.U.)		p16 expression (A.U.)		Inflammatory Score	
			O.R.	*p* value	O.R.	*p* value	O.R.	*p* value	O.R.	*p* value
Motor Impairment	at 36 months^a^		0.217	0.273	1.000	0.698	0.988	0.831	1.006	0.939
	Change over 36 months^b^	Motor Improvement	0.383	0.534	0.415	0.171	0.995	0.805	1.086	0.367
		Motor Impairment	0.535	0.748	1.000	0.941	0.471	0.288	1.110	0.374

O.R., Odds Ratio; ^a^Binary Logistic Regression. ^b^Multinomial Logistic Regression. Reference category: No Change from Baseline to 36 months follow-up; Motor Improvement: from HY≥3 to HY < 3; Motor Impairment: from HY < 3 to HY≥3. All models adjusted for age, gender, baseline LEDD and BMI.

The baseline composite inflammatory score was only predictive of MoCA score (beta = –0.205, *p* = 0.037) at 36 months (Table 4), independently of age, gender, BMI and LEDD, but not for the rate of change for any clinical indicator over the follow-up period. None of our candidate baseline biomarkers of senescence and inflammation significantly predicted MMSE score (Table 4) or motor function (Table 5) either at 36 months or as rate of change over 36 months.

## DISCUSSION

This study included newly diagnosed PD patients at a very early stage of disease, and an age matched control cohort, who were followed up every 18 months to 36 months, and where we had baseline measures of senescence and inflammation. We considered several markers that have been associated with ageing and cellular senescence including telomere length, p21 and p16 expression levels as well as markers of inflammation. Our aim was to test the hypothesis that increased senescence and inflammation at baseline would drive a faster cognitive and motor decline.

### Senescence markers

We measured the association of three potential markers of cell senescence—telomere length, p16 and p21 expression in peripheral blood mononuclear cells (PBMCs) with PD status and progression. We found that telomere length at baseline discriminated well between PD patients and age-matched controls, in line with some previous cross-sectional studies [21–23, 25], but conflicting with others [12, 13, 24, 26]. A previous meta-analysis suggested no telomere length differences overall, although there were large discrepancies on size and gender distributions of the cohorts included as well as in the types of tissues sampled [11]. Nevertheless, we also showed a similar difference at 18 months follow-up, with faster rates of telomere shortening in PD participants. This is in contrast to a recent study [23] that did not find different telomere attrition rates between baseline and 36 months for controls and PD patients. While both PD cohorts were very similar in terms of HY and MDS-UDPRS-III scores at baseline, our cohort was slightly younger (about 3 years), with shorter disease duration at baseline and, importantly, larger. Importantly, we found shorter telomeres at baseline in those PD patients who went on to develop an early dementia. These patients showed no differences in p21 or p16 gene expression levels, or inflammatory score compared to those who did not develop dementia. Unfortunately, the small number of incident PDD cases in our study precluded a meaningful regression analysis of the predictive power of telomere length for PDD. However, the relationship between shorter telomeres in peripheral blood cells and incident dementia has been reported in the general population [19], in stroke [10], AD [54, 55], Down’s syndrome [56] and in the transition from MCI to dementia [57]. Consequently, our results suggest that short telomeres may have predictive potential for the development of PDD.

Our results for p21 and p16 were less in line with expectations. The known role of p21 and p16 in cell senescence suggested their relevance as biomarkers of ageing [37, 58]. However, there are few studies on their links to human neurodegeneration [59]. It has been shown previously that p16 in brain neurons and astrocytes increases with ageing and neurodegeneration in mice and that various other senescence markers increase in neurons of ageing mice [33, 36]. Importantly, eradication of senescent cells in a mouse model for PD resulted in better motor function, suggesting a therapeutic benefit [60]. In addition, regional PD-specific white matter alterations have been correlated with increased peripheral leukocyte apoptosis and systemic inflammation [61].

However, we found no significant differences between PD and controls for p16 expression at any time point and, contrary to expectations, a lower p21 expression level in PD patients at baseline compared to controls. Moreover, PD patients who presented with MCI at recruitment had lower p21 baseline expression levels than those who were cognitively normal. Similarly, low levels of p16 at baseline were robustly associated with faster disease progression. These observations add to existing evidence suggesting that the association of senescence of blood cells with neurodegenerative diseases might not be as simple as originally thought. In particular, our own previous immuno-phenotyping studies showed a reduction of ‘late senescent’ CD8+ T cells (identified as CD28^lo^CD57^hi^) in PD cases versus controls [62]. Furthermore, in AD patients monocytic p21 protein levels were decreased compared to control individuals [59]. Hence it is possible that a more activated, less senescent immune system might contribute to neurodegeneration.

This study was limited in terms of available samples such that we were not able to measure protein levels in PBMCs. A previous small study of p21 protein levels in PBMCs did not find significant differences between PD patients and controls for either total p21 protein or its phosphorylated form p21(Thr145) [63]. However, the authors found that phosphorylated Ser15 p53 and Thr145- phosphorylated p21 proteins were significantly increased in blood samples from 10 AD cases compared to controls. Thus, it is possible that only phosphorylated p21 is increased during certain neurodegenerative diseases, while total p21 protein levels, and perhaps p21 gene expression levels, may be less informative. In addition, p16 (and by extension, p21) mRNA levels in total white blood cells might not be sensitive enough: alterations may only be detectable in separated T lymphocyte populations [64] but we did not have the appropriate samples to investigate this.

### Inflammatory markers

We created a simple (baseline) inflammatory score as a summary variable reflecting the inflammatory status of our participants. This score was significantly higher in PD cases than in controls in agreement with previous results using a principal component analysis within the ICICLE-PD study extended cohort [40] and by others analysing individual markers [61, 65]. Furthermore, our inflammatory score in PD cases at baseline correlated negatively with both cognitive parameters (MMSE and MoCA) and positively with the motor function MDS-UPDRS-III scores at baseline. It was also a significant predictor of both motor (MDS-UPDRS-III) and cognitive (MoCA) function in PD cases at 36-months, in line with a previous study [40]. A very similar composite inflammatory score including TNF-alpha, IL-6 and CRP has already shown to be, after age, the main predictor of cognitive function for those aged over 75 years [52]. Given the wide disparity between studies on individual cytokines [66, 67], such composite variables may offer a more informative evaluation of disease progression in PD [65] and the anti-inflammatory effect of potential treatments [68].

In summary, our study demonstrates that telomere lengths at baseline and 18 months were lower in PD patients compared to age-matched healthy controls with shorter telomere length at baseline and at 18 months also associated with development of dementia within 36 months. A baseline inflammatory score consisting of five different cytokines gave the best prediction for cognitive scores of PD cases 3 years later, while lower p16 gene expression predicted a more rapid disease progression over the same period in relation to both cognitive and motor scores. Thus, both inflammatory and senescence markers (telomere length and p16) warrant further investigation as potential predictors of disease progression in PD.

## CONFLICT OF INTEREST

The authors have declared that there are no competing interests and nothing to disclose.

## Supplementary Material

Supplementary Material
