# Supplementary material for: Senescence and Inflammatory Markers for Predicting Clinical Progression in Parkinson’s Disease: The ICICLE-PD Study
Source: J Parkinsons Dis. 2020 Jan 13;10(1):193–206. doi: 10.3233/JPD-191724 (PMC7029330; doi:10.3233/JPD-191724)
Supplement: Supplementary Material [file jpd-10-jpd191724-s001.docx]

**Supplementary Material**

**Senescence and Inflammatory Markers For Predicting Clinical Progression in Parkinson’s Disease: The ICICLE-PD Study**

**Supplementary Figure 1a.** p21 expression at baseline and 18 months (A) and p21 expression change over 18 months (B) in controls and Parkinson’s disease patients. Each category box plot includes the median ( **̶̶̶̶ ̶̶̶̶̶̶ ̶** ), the range of data within the first and third quartiles (box), the range of data within the first and ninth deciles (whiskers) and the outliers falling outside the latter (° = Outlier, *= Extreme Outlier). ^a^ Statistically significant using the Mann-Whitney U Test.

Baseline 18m Follow-up Baseline 18m Follow-up

p21 Expression change rate (per month)

Control Parkinson’s Disease

N=73 N= 122

**A**

**B**


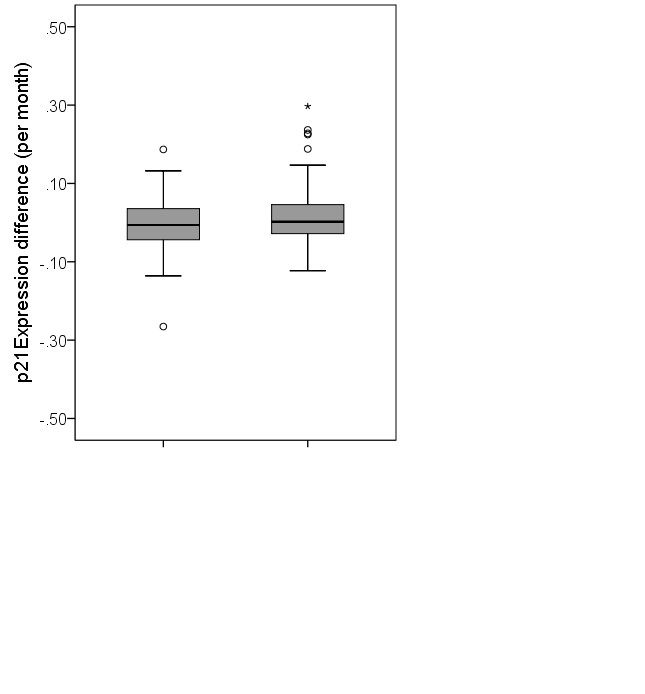


4.00

3.00

2.00

1.00

.00

-1.00

-2.00

p21 Expression (Log)

Control Parkinson’s Disease

N=93 N=77 N= 142 N=126

^a^ p<0.001

#### Supplementary Figure 1b. p16 expression at baseline and 18 months (A) and p16 expression change over 18 months (B) in controls and Parkinson’s disease patients. Each category box plot includes the median ( ̶̶̶̶ ̶̶̶̶̶̶ ̶ ), the range of data within the first and third quartiles (box), the range of data within the first and ninth deciles (whiskers) and the outliers falling outside the latter (° = Outlier, *= Extreme Outlier). ^a^ Statistically significant using the Mann-Whitney U Test.

.50

.40

.30

.20

.10

.00

p16 Expression change rate (per month)

Control Parkinson’s Disease

N=73 N= 122

4.00

3.00

2.00

1.00

.00

-1.00

-2.00

Baseline 18m Follow-up Baseline 18m Follow-up

Control Parkinson’s Disease

N=93 N=77 N= 142 N=126

p16 Expression (Log)

**A**

**B**

#### Supplementary Table 1. Spearman’s rho correlation between baseline biomarkers and clinical outcomes at Baseline and 36 months later (adjusted).

| Control cases^a^ |  | TL (T/S ratio) | | p21 (A.U.) | p16 (A.U.) | CRP (ng/ml) | TNF alpha (pg/ml) | IL-6 (pg/ml) | IL-10 (pg/ml) | IFNg (pg/ml) | | Inflammatory score | |
| --- | --- | --- | --- | --- | --- | --- | --- | --- | --- | --- | --- | --- | --- |
| Baseline MMSE | Corr. coeff | -0.038 | | -0.001 | 0.149 | -0.067 | -0.083 | -0.130 | -0.180 | -0.080 | | -0.180 | |
|  | Sig. | 0.745 | | 0.995 | 0.157 | 0.526 | 0.433 | 0.218 | 0.099 | 0.453 | | 0.100 | |
| Baseline MoCA | Corr. coeff | 0.028 | | -0.119 | -0.078 | -0.034 | -0.079 | 0.112 | -0.284 | -0.039 | | -0.086 | |
|  | Sig. | 0.809 | | 0.263 | 0.458 | 0.749 | 0.456 | 0.289 | **0.008** | 0.711 | | 0.431 | |
| 36months MMSE | Corr. coeff | -0.197 | | 0.035 | 0.282 | -0.091 | -0.018 | -0.161 | 0.099 | 0.211 | | -0.006 | |
|  | Sig. | 0.116 | | 0.780 | **0.020** | 0.463 | 0.886 | 0.194 | 0.435 | 0.087 | | 0.962 | |
| 36months MoCA | Corr. coeff | 0.066 | | 0.033 | 0.086 | 0.021 | 0.010 | 0.168 | -0.041 | 0.234 | | 0.021 | |
|  | Sig. | 0.602 | | 0.788 | 0.487 | 0.865 | 0.934 | 0.174 | 0.751 | 0.056 | | 0.867 | |
| Parkinson's disease^b^ |  |  |  | | | | | | | |  | |  |
| UPDRS-III Baseline | Corr. coeff | -0.044 | | 0.051 | -0.026 | 0.062 | 0.154 | 0.169 | 0.110 | 0.080 | | 0.248 | |
|  | Sig. | 0.632 | | 0.545 | 0.762 | 0.477 | 0.078 | 0.054 | 0.222 | 0.361 | | **0.005** | |
| Baseline MMSE | Corr. coeff | 0.140 | | 0.162 | -0.084 | -0.100 | -0.210 | -0.139 | -0.063 | -0.158 | | -0.191 | |
|  | Sig. | 0.130 | | 0.055 | 0.320 | 0.255 | **0.016** | 0.116 | 0.483 | 0.071 | | **0.033** | |
| Baseline MoCA | Corr. coeff | 0.009 | | 0.189 | -0.005 | -0.125 | -0.237 | -0.163 | -0.023 | -0.134 | | -0.208 | |
|  | Sig. | 0.925 | | **0.032** | 0.954 | 0.179 | **0.010** | 0.081 | 0.807 | 0.149 | | **0.028** | |
| 36months UPDRS-III | Corr. coeff | -0.074 | | 0.021 | -0.160 | 0.297 | 0.064 | 0.197 | -0.106 | 0.090 | | 0.184 | |
|  | Sig. | 0.469 | | 0.836 | 0.104 | **0.003** | 0.534 | 0.054 | 0.311 | 0.382 | | 0.079 | |
| 36months MMSE | Corr. coeff | 0.020 | | 0.125 | 0.170 | 0.018 | -0.197 | -0.193 | 0.088 | -0.045 | | -0.114 | |
|  | Sig. | 0.843 | | 0.208 | 0.084 | 0.864 | 0.053 | 0.060 | 0.403 | 0.662 | | 0.280 | |
| 36months MoCA | Corr. coeff | 0.070 | | 0.148 | 0.097 | -0.162 | -0.238 | -0.324 | 0.061 | -0.093 | | -0.287 | |
|  | Sig. | 0.496 | | 0.140 | 0.336 | 0.114 | **0.020** | **0.001** | 0.567 | 0.367 | | **0.006** | |
| ^a^ Adjusted for age and gender; ^b^ Adjusted for age, gender and baseline LEDD. Corr. Coeff, Spearmann’s rho correlation coefficient; Sig., Significance (2-tail). | | | | | | | | | | | | | |
